# Achieving surface chemical and morphologic alterations on tantalum by plasma electrolytic oxidation

**DOI:** 10.1186/s40729-016-0046-2

**Published:** 2016-04-16

**Authors:** Marcelo Augusto Pinto Cardoso Goularte, Gustavo Frainer Barbosa, Nilson Cristino da Cruz, Luciana Mayumi Hirakata

**Affiliations:** 1 Department of Prosthodontics, Implantology Pontifical Catholic University of Rio Grande do Sul - PUCRS, Av. Ipiranga, 6681 Prédio 06, Partenon, CEP: 90619-900, Porto Alegre, RS Brazil; 2 Clinical Department, Universidade Luterana do Brasil - Torres (ULBRA-TORRES), Rua Universitária, 1900, Parque do Balonismo, CEP 95560-000, Torres, RS Brazil; 3 Department of Engineering, Universidade Estadual Paulista Júlio de Mesquita Filho, UNESP, Av. 3 de março, 511, Alto da Boa Vista, CEP: 18087-180 Sorocaba, SP Brazil; 4 Department of Dental Materials, Pontifícia Universidade Católica do Rio Grande do Sul - PUCRS, Av. Ipiranga, 6681 Prédio 06, Partenon, CEP: 90619-900 Porto Alegre, RS Brazil

**Keywords:** Tantalum, Implant surface treatment, Plasma electrolytic oxidation, Biomaterials

## Abstract

**Background:**

Search for materials that may either replace titanium dental implants or constitute an alternative as a new dental implant material has been widely studied. As well, the search for optimum biocompatible metal surfaces remains crucial. So, the aim of this work is to develop an oxidized surface layer on tantalum using plasma electrolytic oxidation (PEO) similar to those existing on oral implants been marketed today.

**Methods:**

Cleaned tantalum samples were divided into group 1 (control) and groups 2, 3, and 4 (treated by PEO for 1, 3, and 5 min, respectively). An electrolytic solution diluted in 1-L deionized water was used for the anodizing process. Then, samples were washed with anhydrous ethyl alcohol and dried in the open air. For complete anodic treatment disposal, the samples were immersed in acetone altogether, taken to the ultrasonic tank for 10 min, washed again in distilled water, and finally air-dried. For the scanning electron microscopy (SEM) analysis, all samples were previously coated with gold; the salt deposition analysis was conducted with an energy-dispersive X-ray spectroscopy (EDS) system integrated with the SEM unit.

**Results:**

SEM images confirmed the changes on tantalum strips surface according to different exposure times while EDS analysis confirmed increased salt deposition as exposure time to the anodizing process also increased.

**Conclusions:**

PEO was able to produce both surface alteration and salt deposition on tantalum strips similar to those existing on oral implants been marketed today.

## Background

The use of materials that come into direct contact with human tissues such as the bone requires maximum biological security. These materials remain for a long period of time or even indefinitely in the human body, and no negative reactions, like toxicity or carcinogenic effects, shall be acceptable.

For this reason, biocompatibility of new materials has been widely studied, and only after a lot of testing, they can become ready for use in biomedical areas. Titanium is one of these materials, and it is used for implant applications due to its favorable weight-to-strength ratio and good biological performance in the bone, which is intimately dependent on surface properties such as surface roughness, surface chemistry, and wettability [1]. Such features of titanium have led researchers from many different fields to seek alternative materials. As a result, in recent years, a lot of progress has paved the way to creating innovative biomaterials in order to better existing treatments and develop new ones for improved quality of life of patients [2]. One of these materials that may either replace titanium dental implants or constitute an alternative as a new dental implant material is tantalum. This metal was first used for dental implants in 1962. However, problems with costs, metallurgical processes, and poor design have left this material in the background. Today, because of its biocompatible properties and biomechanical qualities associated with new production processes, newly obtained sources, and new dental implant designs, a growing interest in its use in implant dentistry has developed [3].

At the same time, the crucial search for the best biocompatible metal surface has led to the development of surface treatments that aim to create an ideal topography for cell proliferation, protein adhesion, and better mineral salt deposition [4–6] on titanium dental implants. In order to achieve this purpose, a large number of methods have been used over the last decade to change dental implant surface texture, including grit blasting, acid etching, and anodization [7]. One of these processes is plasma electrolytic oxidation (PEO), also known as micro-arc oxidation (MAO) or anodic spark deposition (ASD). This process was slightly modified in 2000 when the TiUnite™ dental implant surface was introduced. The results were very satisfactory [8, 9], and now, TiUnite™ is the major surface treatment applied on titanium dental implant patterns.

In this way and following the successful results already obtained with Titanium, this study aimed to develop an oxidized surface layer on Tantalum samples and, subsequently, analyze the samples’ topography and levels of salt deposition using an electronic microscope.

## Methods

### Tantalum

We used 60 strip-shaped samples of tantalum with the following dimensions: 7 mm wide, 11 mm long, and 0.01 mm thick (Kurt J. Lesker Company—USA, 99.95 % purity). The samples were washed in distilled water and placed in an ultrasonic tank containing acetone (Ultra Sonic-1440 Plus—Odontobrás, Ribeirão Preto/SP, Brazil) to remove residues. Then, they were divided into four groups: in group 1 (control), tantalum received no treatment; in group 2, strips of tantalum were treated using PEO for 1 min; in group 3, tantalum strips were treated using PEO for a 3-min exposure; and in group 4, tantalum strips were treated using PEO for a 5-min exposure. This is shown in Table 1.

**Table 1:** Distribution of groups

Groups	Plasma electrolytic oxidation—time (min)	Voltage (V)	Current (A)
1	–	–	
2	1	ΔU = 160 to 200 V	≅0.18
3	3	ΔU = 160 to 280 V	≅0.19
4	5	ΔU = 160 to 300 V	≅0.18

Then, the samples were washed with anhydrous ethyl alcohol (99.3° INPM, BM Anhydrous Alcohol Cycle, Serrana/SP).

### Anodizing process

A self-organized porous surface of tantalum (Ta) was obtained through oxide formation of Ta using the PEO process. The anodizing process was conducted using an electrolytic solution containing 0.2 mol calcium acetate Ca (CH_3_CO_2_)_2_ H_2_O and 0.02 mol sodium glycerophosphate (hydrated salt) C_3_H_7_Na_2_O_6_P diluted in 1-L deionized water [10–13].

Following Yerokhin [14], in order to perform the anodizing process, Ta sample surfaces were previously cleaned in ethanol and distilled water and then air-jet dried. Then, the samples were immersed in the electrolyte solution and connected to an open circuit, where Ta was the anode (connected to the positive pole), and to a platinum plate functioning as a cathode (connected to the negative pole). Samples were treated in a reactor, driven by an electric system consisting of the following components: AC power source with variable output voltage, a transformer, a rectifying circuit, a circuit breaker, an ammeter, and a voltmeter. An oscilloscope was used to verify the waveform after rectification [12]. The processing system is composed of the electrode support and the electrolyte tank [12]. During treatment, the temperature of the electrolytic solution was measured by a portable thermometer.

Within a 50-mL tank, the electrolytic solution as described above received a voltage variation of 160 V initial tension at zero time and a final tension at the preset end-time for each group of samples. There was a gradual increase in voltage due to the maintenance of a fairly constant current at around 0.15 to 0.25 A. The electrolytic solution was periodically changed to prevent solution saturation. In group 2, the solution was changed every four anodizing processes, namely every four treated samples; in group 3, the solution was changed every two anodizing processes, namely every two treated samples; in group 4, the solution was changed every anodizing process, that is, every one treated sample. The experiment was conducted at room temperature.

Following completion of the anodizing process, the samples were quickly removed from the solution, washed with distilled water, and dried in open air. For a complete disposal of the anodic treatment, the samples were immersed in acetone altogether (Lot PA-55.317- Delaware Supplier, Porto Alegre/RS, Brazil) and taken to the ultrasonic tank (Ultra Sonic-1440 Plus—Odontobrás, Ribeirão Preto/SP, Brazil) for 10 min, washed again in distilled water, and finally air-dried.

### Scanning electron microscopy

All samples were coated with gold prior to scanning electron microscopy (SEM), which was performed with an EVO-LS15 (Zeiss). Observations were made at magnifications between ×500 and ×10.000 and limited to 20 μm for the ×500 and ×1.000 magnifications and to 2 μm for the ×5.000 and ×10.000 magnifications.

### Analysis of salt deposition

The analysis of salt deposition on the samples, occurring during the anodizing process, was performed using the energy-dispersive X-ray spectroscopy (EDS) system (energy-dispersive X-ray detector (EDD) or EDX), which is integrated with scanning electron microscopy unit.

## Results

### Surface treatment

**Fig. 1:**
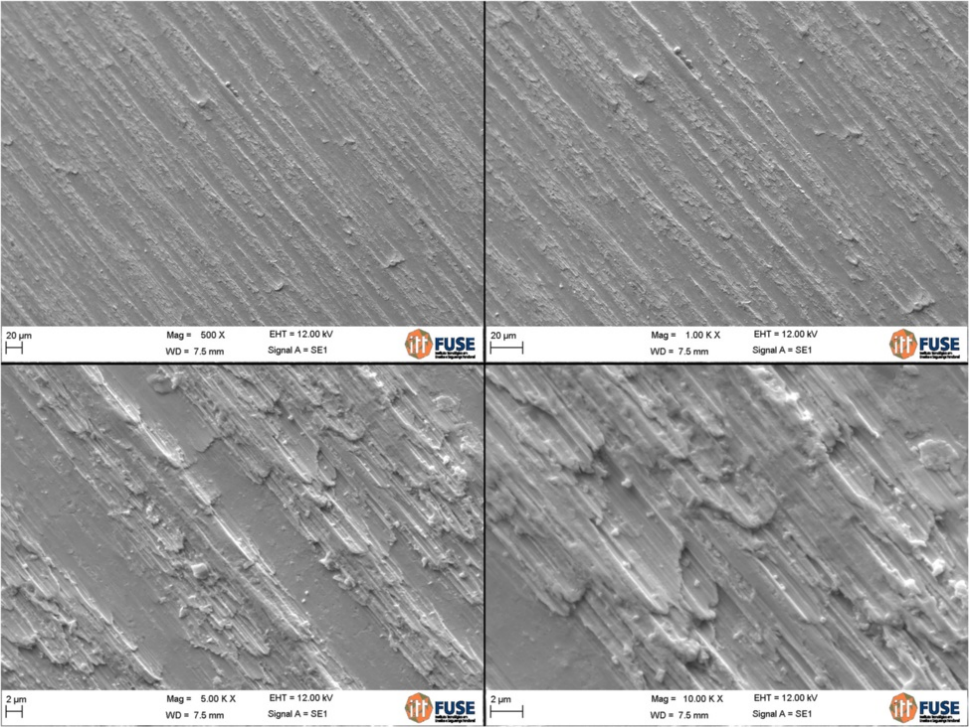
Group control

**Fig. 2:**
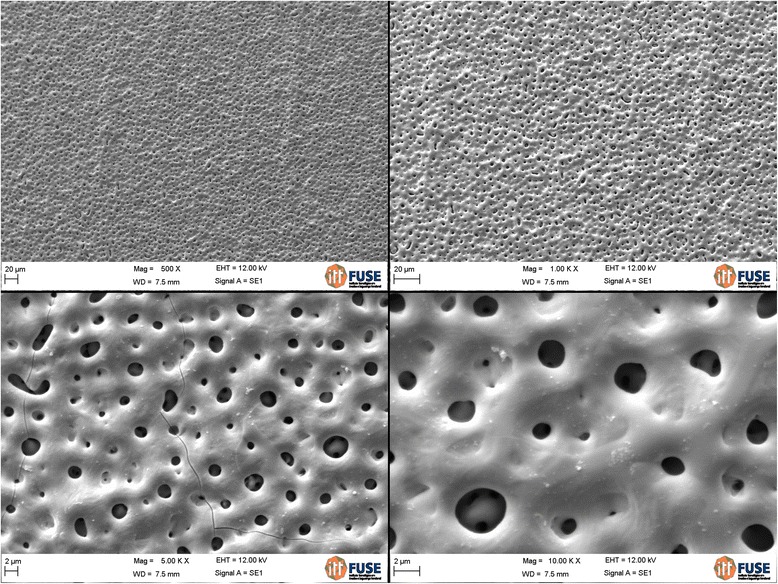
Group 2—1 min

**Fig. 3:**
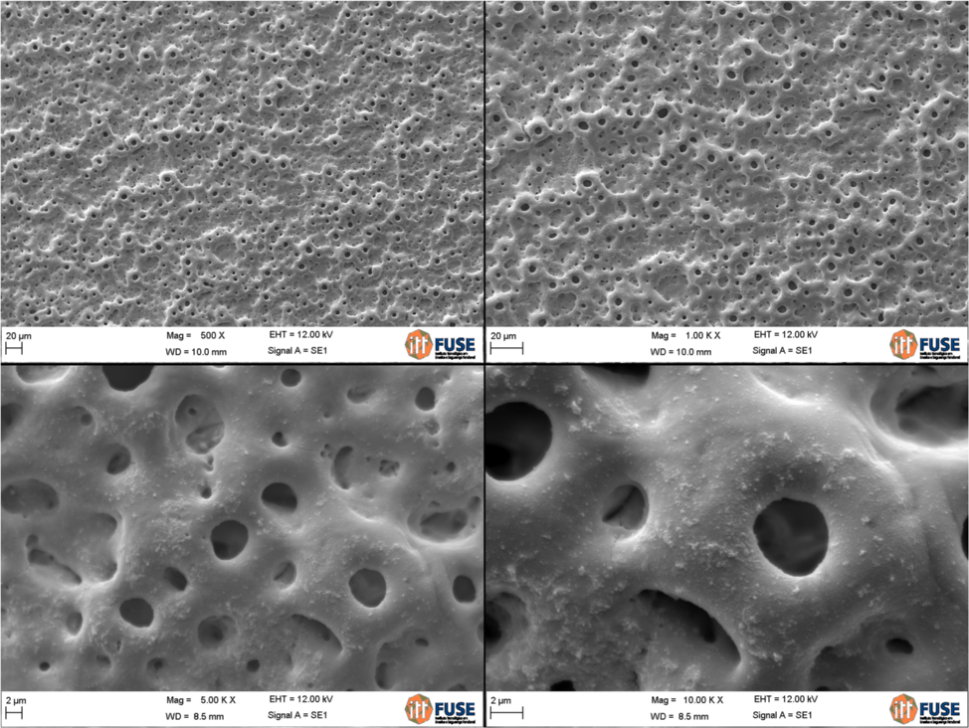
Group 3—3 min

**Fig. 4:**
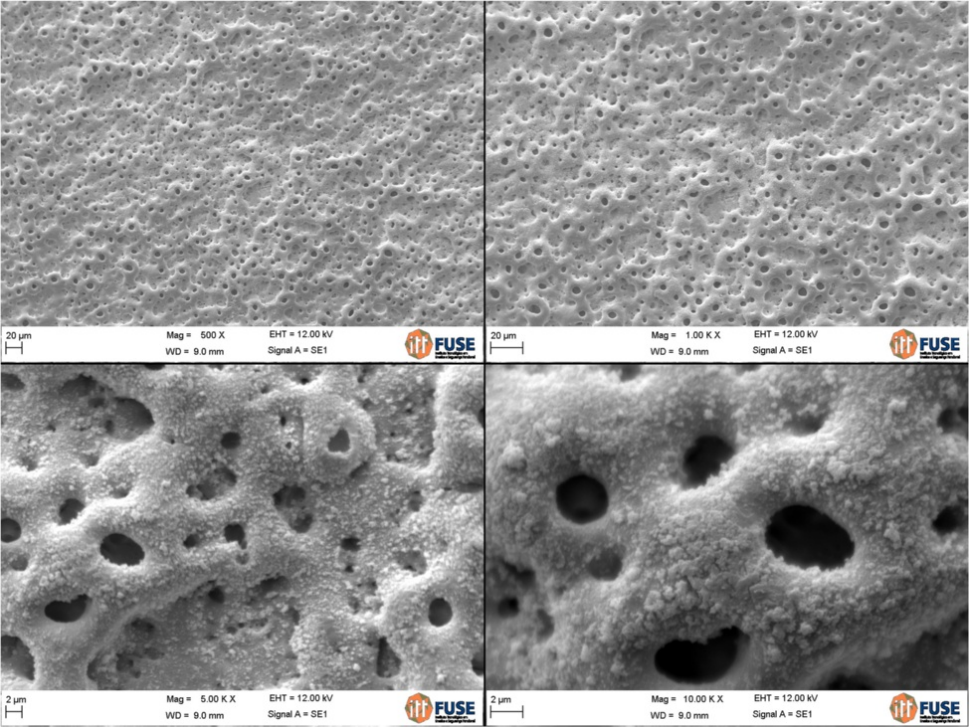
Group 4—5 min

The analysis of the images obtained by scanning electron microscopy confirmed the changes on the surface of tantalum strips according to different exposure times. In Fig. 1, we can observe Ta surface with grooves resulting from the machining of the metal with no surface treatment. As the magnitude increases, the image shows the lines pattern with its peculiar characteristics from the manufacturing of the tantalum strip. In Fig. 2, regular pores can be seen all over the Ta surface after a 1-min anodizing period (group 2). In addition, it is possible to observe the formation of peaks and valleys of small amplitude, creating an image of a slightly smaller increase in the roughened surface. As we increase the magnitude of the images, we notice the presence of whitish spots, which are salt deposits resulting from oxidation. In Fig. 3, on a 3-min exposure period sample (group 3), changes in the topography can be observed. In addition to the holes, there are both deeper and higher areas. Salt deposition has increased with increasing exposure. In Fig. 4, the surface shows many changes (5-min exposure). Peaks and valleys are quite visible, and salt deposition has spread all over the surface; the topography, however, seems to remain the same as in the previous pattern, with a 3-min exposure period.

### Analysis of salt deposition

EDS analysis of the samples confirmed the increased salt deposition as the exposure time to the anodizing process also increased. The main chemical elements found on the surface of the oxidized tantalum surface were those ones which made up the PEO solution (0.2 mol acetate calcium—Ca (CH_3_CO_2_) _2_H_2_O and 0.02 mol of sodium glycerophosphate—C_3_H_7_Na_2_O_6_P). Tables 2, 3, 4, 5, 6, 7, 8, and 9 show the weight ratios and the atoms of the elements that make up two given points (spectrum) of the field. These points were randomly chosen in the sample.

**Table 2:** Chemical analysis of surface (group 1 spectrum 1)

Element	Weight %	Atoms %
Carbon	8.22	37.21
Oxygen	11.37	38.63
Tantalum	80.41	24.16
Total	100	100

**Table 3:** Chemical analysis of surface (group 1 spectrum 2)

Element	Weight %	Atoms %
Carbon	8.02	38.2
Oxygen	10.19	36.25
Tantalum	81.79	25.55
Total	100	100

**Table 4:** Chemical analysis of surface (group 2 spectrum 1)

Element	Weight %	Atoms %
Carbon	4.47	19.93
Oxygen	15.79	52.89
Calcium	3.41	4.56
Tantalum	76.33	22.62
Total	100	100

**Table 5:** Chemical analysis of surface (group 2 spectrum 2)

Element	Weight %	Atoms %
Carbon	8.54	31.34
Oxygen	16.96	46.73
Calcium	4.43	4.87
Tantalum	70.07	17.06
Total	100	100

**Table 6:** Chemical analysis of surface (group 3 spectrum 1)

Element	Weight %	Atoms %
Carbon	4.79	18.86
Oxygen	16.05	47.44
Sodium	0.53	1.10
Magnesium	1.36	2.63
Calcium	10.64	12.56
Tantalum	66.63	17.41
Total	100	100

**Table 7:** Chemical analysis of surface (group 3 spectrum 2)

Element	Weight %	Atoms %
Carbon	4.12	15.29
Oxygen	20.34	56.78
Sodium	–	–
Magnesium	1.06	1.94
Calcium	8.78	9.78
Tantalum	65.70	16.21
Total	100	100

**Table 8:** Chemical analysis of surface (group 4 spectrum 1)

Element	Weight %	Atoms %
Carbon	10.43	23.42
Oxygen	35.02	59.04
Sodium	–	–
Magnesium	1.12	1.24
Phosphorus	3.68	3.20
Calcium	10.82	7.29
Tantalum	38.93	5.81
Total	100	100

**Table 9:** Chemical analysis of surface (group 4 spectrum 2)

Element	Weight %	Atoms %
Carbon	15.07	26.19
Oxygen	45.97	59.99
Sodium	0.58	0.52
Magnesium	1.38	1.18
Phosphorus	4.51	3.04
Calcium	13.12	6.84
Tantalum	19.37	2.24
Total	100	100

**Fig. 5:**
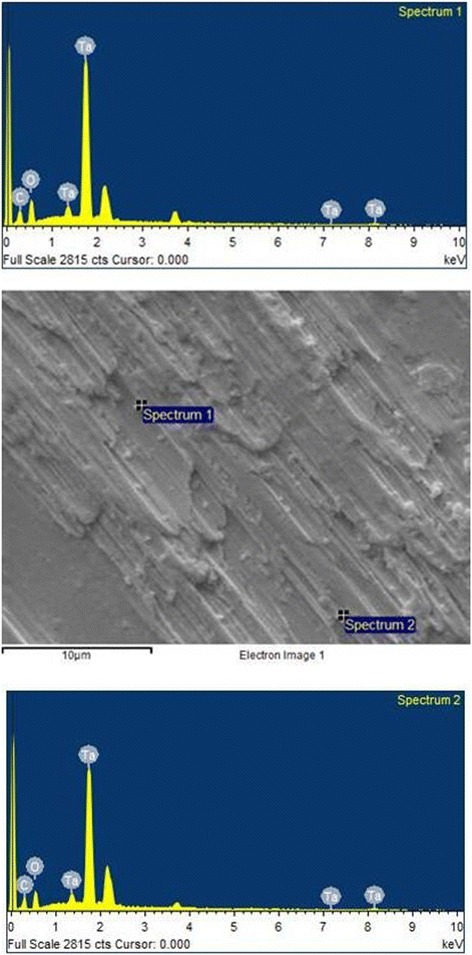
EDS control

**Fig. 6:**
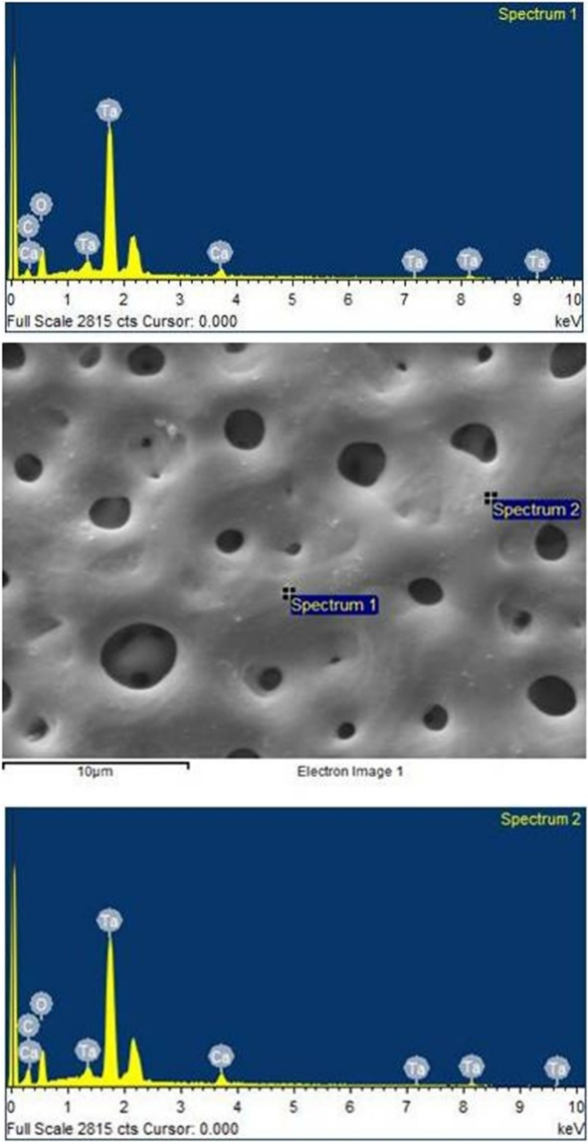
EDS 1 min

**Fig. 7:**
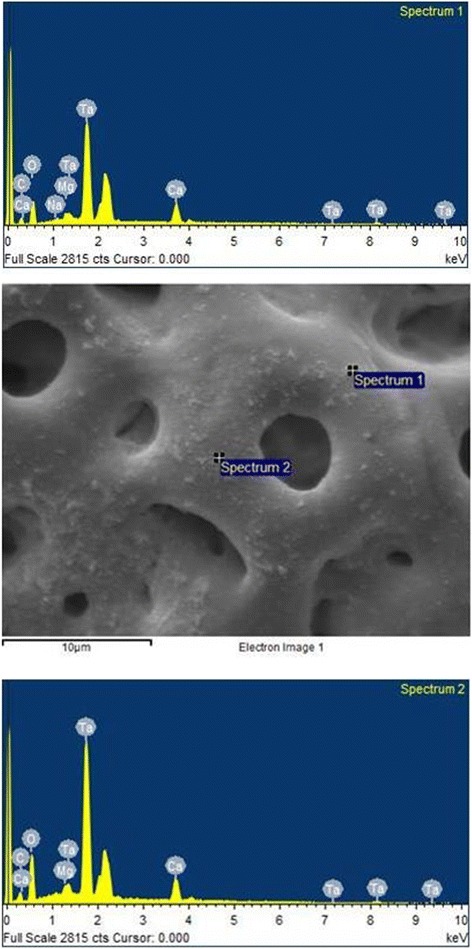
EDS 3 min

**Fig. 8:**
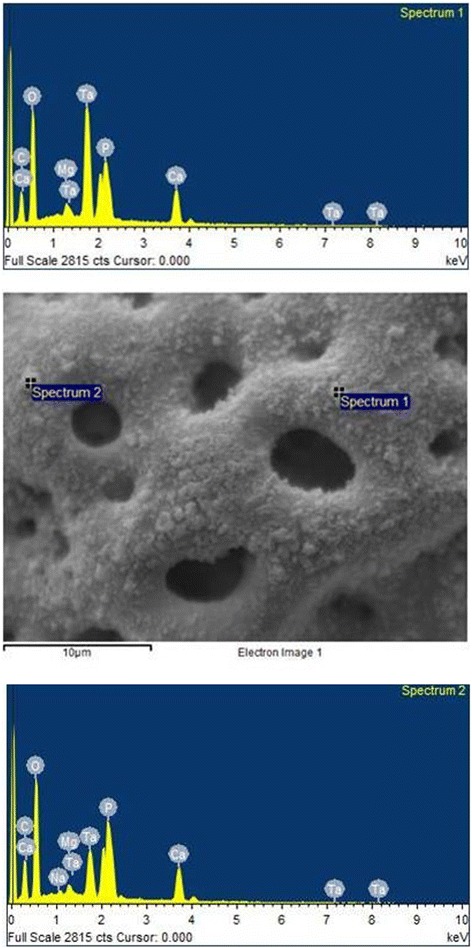
EDS 5 min

Tables 2 and 3 show similar rates among the chemical elements present on the non-treated tantalum surface—group 1 (Fig. 5). In Tables 4 and 5 (group 2), calcium (Ca) is included. The rates for the other chemical elements are similar to the rates in group 1 (Fig. 6). In Tables 6 and 7, group 3 sample shows the basic chemicals present in previous groups and similar rates (Fig. 7). Two chemical elements, magnesium (Mg) and small quantities of sodium (Na), are included. There is a decrease in tantalum (Ta) rate in the sample. Tables 8 and 9 show that the chemical element rates are similar between the two spectra of group 4 samples (Fig. 8). Another chemical element is included: phosphorus (P). Tantalum rates decrease whereas the oxidation layer increases.

## Discussion and conclusions

The search for new biomaterials and biocompatible metals has always been a common objective of human rehabilitation research centers. In implant dentistry, titanium has successfully established itself as the material of choice for dental implants. However, several studies have reported cases of metal allergy caused by titanium-containing materials [15–17] and some immune dysfunctions in certain patients chronically exposed to this reactive metal [17]. Because of such constraints, research has turned once again to tantalum, a high biocompatible, inert, corrosion resistant metal that began to be studied in the [3] 1940s. Implantology research became interested in this metal in the 1960s, but the feedback from implant dentistry was not positive at all. Factors such as high costs and implant design prevented tantalum from being widely accepted, and, therefore, its use was not as successful as titanium’s.

Interestingly, despite limited tantalum use in orthopedic implant devices [3], this metal has been used for prostheses in Medical Orthopedics until today. In implant dentistry specifically, the role of tantalum has been changing due to certain factors. Firstly, its price is no longer a constraint because the demand for this metal in other technologies has increased, and new source areas were discovered like, for example, the tantalum ore in Brazil in 2008, which proved to be the biggest reserve of tantalum in the world [18]. Secondly, the low success rate reached previously is related to the fact that tantalum was the pioneer metal for implantology and it was, consequently, affected by the burden of innovation. The first tantalum dental implants did not have appropriate stability, and at that time, the knowledge about factors associated with good implant installation procedures and biomechanical aspects was not as advanced as it is today. The process of osseointegration, for example, was first described by Professor Bränemark only in 1977 [19]. Thirdly, there is a recent trend in research and development of titanium alloys specifically for biomedical applications that addresses concerns with toxic effects of the dissolution of aluminum and vanadium ions into the host tissue as a result of corrosion wear of titanium alloy (Ti6Al4V) [2]. In addition, chemical inertness and biocompatibility of Ta, similar to titanium’s and its oxides, as a result of Ta oxides forming on the surface of Ta, add positively to the abovementioned factors. An oxide layer of Ta can form on the metal surface immediately after the surface is exposed to oxygen, because Ta is highly reactive to oxygen [3].

Thus, this study has examined the possibility of activating an alteration surface in tantalum using the anodizing process, which is effective in other metals like titanium. From our findings, it was possible to develop time exposure protocols in order to obtain conductive surface alterations similar to those already available from some of the largest manufacturers of oral implants. Scanning electron microscopy analyses showed satisfactory topography patterns, very similar to the ones occurring on titanium surfaces. In addition, the salt deposition analyses showed an increased oxidation layer as exposure time increased. In contrast, the presence of tantalum on the strip surface decreased as the oxidation layer increased in each group. Also, other chemical elements from the electrolytic solution added to the strip surface as exposure time and electrical potential increased on the samples’ oxidation layer.

Despite these promising outcomes, the optimum composition of a chemically oxidized surface layer and the necessary chemical elements for good bone formation and good bone adherence are still unknown. Extensive physical and chemical characterization of these surfaces has been described in the literature, but in vitro biological responses to them have not been clarified yet [6]. Therefore, knowing the in vitro study limitations, we strongly recommend further investigations in order to establish the optimum protocol, learn about the ideal surface composition of tantalum, and better understand cytotoxicity and bone bioactivity.
